# Haploidentical Hematopoietic Stem Cell Transplantation for Patients with Severe Aplastic Anemia—Single-Centre Experience

**DOI:** 10.3390/curroncol31030093

**Published:** 2024-02-26

**Authors:** Vered Stavi, Niranjan Khaire, Jeffrey H. Lipton, Rajat Kumar

**Affiliations:** 1 Soroka Medical Center, Beer Sheva 84101, Israel; vered.stavi@gmail.com; 2 Princess Margaret Cancer Centre, Toronto, ON M5G 2M9, Canada; niranjankhaire@gmail.com (N.K.); jeff.lipton@uhn.ca (J.H.L.)

**Keywords:** severe aplastic anemia, allogeneic bone marrow transplant, haploidentical donor

## Abstract

Severe aplastic anemia (SAA) is a life-threatening type of aplastic anemia for which allogeneic stem cell transplantation or immunosuppressive therapy are the principal treatment modalities. Only about 25–30% of patients have a matched sibling donor, and finding an unrelated donor in ethnic minorities is a challenge. The use of related haploidentical donor transplants in severe aplastic anemia is uncommon. We would like to report our experience with the first four patients who underwent haploidentical transplants for severe aplastic anemia. This is a retrospective study. We collected data from our transplant database of all haploidentical hematopoietic stem cell transplants for SAA from 1 January 2020 to 31 December 2021. The transplant protocol used was the Hopkins’ protocol. There were three patients who underwent haploidentical transplants as primary therapy for SAA. A fourth patient received a haploidentical transplant after immunosuppressive therapy failure. The median age of the patients at transplant was 24 y (range 20–29). All patients were engrafted. Neutrophil engraftment occurred at a median of 21 days (range 17–22). Any active infections resolved with the recovery of blood counts. The median hospitalization time was 27 days (range 22–41). Only one patient had grade 2 acute GVHD involving the skin. There was no chronic GVHD. All patients had complete lymphoid and myeloid donor chimerism on day 60. Based on our experience and the emerging literature, haplo-identical transplantation should be considered for select young patients with SAA who have low chances of responding to immunosuppressive therapy.

## 1. Background

Aplastic anemia (AA) is a rare but potentially fatal disorder characterized by hypocellular bone marrow and pancytopenia in the absence of marrow infiltration or fibrosis. Severe aplastic anemia (SAA) is a life-threatening category of AA characterized by at least two of the following three criteria: (a) neutrophil count < 0.5 × 10^9^/L, (b) platelet count < 20 × 10^9^/L, and (c) reticulocyte count < 20 × 10^9^/L [1]. For SAA, allogeneic hematopoietic stem cell transplantation (HSCT) and immunosuppressive therapy (IST) are the principal treatment modalities [2]. Very severe aplastic anemia (VSAA) is another subtype of AA, defined as SAA with neutrophils < 0.2 [1]. VSAA has a poor prognosis.

The disease is more common in Asia and among ethnic minorities. A study from Canada showed that the incidence of the disease is higher in ethnic minorities [3]. Upfront matched sibling donor (MSD) HSCT is recommended for patients with SAA who are younger than 40 years of age [1] and reportedly results in long-term survival rates of over 80% [1,2,4]. Unfortunately, only about 25–30% of patients have a matched sibling donor.

According to current therapeutic algorithms, IST using a combination of horse antithymocyte globulin (ATG), cyclosporin A (CsA), and eltrombopag (EPAG) is the preferred first-line treatment for patients without an MSD, and transplantation from a matched unrelated donor (MUD) should be delayed until one or two courses of IST have failed [1,2]. However, approximately one third of patients do not respond to IST, and one third of responders relapse after initial therapy. Moreover, 10–15% of patients treated with IST evolve a clonal evolution [2,5]. There is an emerging opinion among experts that offering upfront MUD transplants to young patients with SAA, especially if they have uncontrolled infections, could be beneficial [6,7]. However, finding a MUD in ethnic minorities is a challenge, as they are not well represented in donor registries [8].

In recent years, the use of a related haploidentical donor for HSCT for those who do not have an MSD or MUD has increased in the treatment of hematological malignancies. However, the use of related haploidentical donor transplants in SAA is uncommon. While this method has been used in China for many years [9], due to the lack of matched related siblings resulting from the single child norm, there is very limited experience in other parts of the world. Moreover, the protocol used in most of the centres in China is complex, as it includes the administration of busulfan, and the graft source is a combination of granulocyte colony-stimulating factor (G-CSF)-primed bone marrow and peripheral blood stem cells (PBSCs) [10]. While survival is good, the incidence of graft versus host disease (GVHD) has been high, with acute GVHD graded II-IV at 43.9%, limited chronic GVHD at 47.7%, and extensive chronic GVHD at 12.1% [10]. Recently, with the use of post-transplant cyclophosphamide (PTCY), haplo-identical transplants have been performed in the Western world but are still considered experimental [2,11]. DeZern et al. have shown very encouraging results with haplo-transplants in SAA using PTCY [12]. In their initial study, the results were very good, with an overall survival of 94% at 1 and 2 years and an incidence of grade II-IV acute GVHD at day 100 of 11% and chronic GVHD at 2 years of 8% [12].

One of the advantages of a haploidentical transplant is that almost all patients will have a related haploidentical donor, as a patient would share one inherited haplotype with a parent or a child and half of the siblings. Hence, most patients will be eligible for this curative therapy. Moreover, these transplants can be performed urgently, as family members are generally available and motivated. We would like to report our experience with the first four patients who underwent haploidentical HSCT for SAA at our centre.

## 2. Methods

This is a retrospective study. We collected data from our transplant database of all haploidentical hematopoietic cell transplants for SAA from 1 January 2020 to 31 December 2021.

Institutional research board approval of the retrospective data collection and analysis was obtained (22-5109, 13 June 2022, renewed May 2023). Informed consent was obtained from all patients.

The transplant protocol used was the Hopkins’ protocol [12]. In summary, the protocol consisted of the following:

Rabbit ATG was dosed at 0.5 mg/kg on day -9 and 2 mg/kg on days -8 and -7. A total of 30 mg/m^2^ of Fludarabine was administered IV daily from day -6 to day -2. A total of 14.5 mg/kg of Cyclophosphamide was administered IV daily from day -6 to day -5, and total body irradiation (TBI) was performed with a single nonmyeloablative dose of 200 cGy on day 1 in the salvage transplants and 400cGy in the upfront transplants.

G-CSF was administered on day +5 at 5 mcg/kg per day until neutrophil engraftment, defined as absolute neutrophil count > 0.5 × 10^9^/L. The GVHD prophylaxis comprised ATG (also part of conditioning), along with (a) PTCY administered at 50 mg/kg per day IV on days +3 and +4, (b) Mycophenolate mofetil from days 5 through 35, and (c) Cyclosporine from day +5 onwards to approximately one year. We used cyclosporine instead of tacrolimus, which was used in the original Hopkins’ protocol.

All patients were admitted to the transplant unit in individual rooms with HEPA filters. They were given prophylactic antibacterial cover with ciprofloxacin until neutrophil engraftment. For fungal prophylaxis, caspofungin was administered, and after engraftment, it was replaced by posaconazole, which was continued until day 100 post-transplant. Letermovir was given for CMV prophylaxis until day 100. Prophylaxis for Pneumocystis jirovecii pneumonia (PJP) was given for one year after transplant with trimethoprim–sulfamethoxazole or inhaled pentamidine. Prophylactic valacylovir was administered for one year post-transplant. All patients were immunized as per the transplant protocol.

## 3. Results

There were three patients who underwent haploidentical transplants as primary therapy for SAA, termed as upfront transplant. They had not received prior ATG. A fourth patient (patient #2) received a haploidentical transplant after immunosuppressive therapy failure, termed as salvage haploidentical transplant. This patient (#2) had received ATG twice, as well as multiple immunosuppressive agents and EPAG, but remained unresponsive to treatment. Further details on these patients are given in Table 1.

None of these patients had a matched sibling or unrelated donor. All of them self-identified as non-white. No patient had donor-specific antibodies against their chosen haplo-identical donors. The graft source was bone marrow in all the three patients who received an upfront transplant. In the patient with a salvage transplant (patient #2), the graft source was G-CSF-stimulated peripheral blood stem cells, in order to facilitate early neutrophil engraftment. In this patient, there was a concern regarding risk of graft rejection, as this patient had received multiple blood transfusions for her aplastic anemia.

The median age of the patients at transplant was 24 y (range 20–29). All patients were engrafted. Neutrophil engraftment occurred at a median of 21 days (range 17–22). Any active infections resolved with the recovery of blood counts. The median hospitalization time was 27 days (range 22–41). Only one patient had grade 2 acute GVHD involving the skin. There was no chronic GVHD. All patients had complete lymphoid and myeloid donor chimerism on D+60.

One patient (#1) developed poor graft function after CMV viremia, which required treatment with valgancylovir. The cytopenias resolved after the addition of EPAG treatment, which was given for ten months. EPAG was well tolerated, with no adverse effects. Two patients developed hemorrhagic cystitis, and one required readmission to manage this complication. BK virus was positive in the urine of both patients. The hemorrhagic cystitis resolved with supportive therapy, and anti-viral treatment was not required. Only one patient experienced acute GVHD of the skin, grade 2, which resolved with the addition of topical steroids. Three of the patients regained normal menstrual cycles. Two of these patients became pregnant, and one gave birth to a healthy baby. All the patients have resumed their normal life and activities. One year on, they are off all medications and immunosuppressive therapy.

## 4. Discussion

In SAA, the only curative treatment is HSCT, whether upfront or as a second line [2]. In young patients or those with very severe aplastic anemia or uncontrolled infections, HSCT appears superior to ATG with or without EPAG [6,7]. In patients with severe or uncontrolled infections, the recovery of neutrophil counts is critical for recovery. In an allogeneic hematopoietic stem cell transplantation, neutrophil recovery generally occurs within two to three weeks after the infusion of the donor stem cells. Hence, the need for hospitalization and intensive care is short and predictable. It is important to use transplant protocols with rapid and successful engraftment, low risk of graft rejection or failure, and low incidence of GVHD. The protocols outlined by the Hopkins’ group fulfill these requirements [12,13].

In contrast, immunosuppressive therapy takes three to six months for a hematologic response. In a patient with active infection, an unpredictable response after many weeks or months is not a good outcome.

The addition of EPAG to ATG and cyclosporine has improved the rate and rapidity of hematologic response compared to standard IST, but overall survival has not improved. Moreover, the clonal evolution rate does not change with the addition of EPAG, remaining at about 15%. Very severe aplastic anemia was found to be a negative predictor for both complete response at 3 months and an overall response at 6 months [14].

These factors favor a hematopoietic cell transplant over IST in those with VSAA or those with active, uncontrolled infections. This poses a challenge in patients who do not have a matched sibling donor. A matched unrelated donor is the next recommended option [1]. The probability of finding a matched unrelated donor depends on the ethnic origin of the patient. The large majority of donors registered in donor registries are of Western European ancestry [15].

Aplastic anemia is more common in ethnic minorities, and with increasing immigration, we may see more patients with severe aplastic anemia in Canada and Western countries. For those who do not have an MSD, the search for a MUD donor is usually unsuccessful. Most of these patients experience repeated cycles of ATG, CsA, blood transfusions, and hospitalizations. Their illness is often prolonged, as seen in patient #2 in our study. Moreover, while the addition of EPAG does result in a faster hematological response to ATG, the long-term complications of relapse and clonal evolution still remain a problem [16].

Unrelated cord blood transplantations (UCBTs) might offer an option for those who do not have an MSD and MUD. The main requirement for a successful cord blood transplant for SAA is an adequate cell dose, which is a limitation for most adults [17]. A phase II study showed that, in refractory SAA patients who had failed at least one course of IST, there was a 2-year survival of 84.6% [18]

Haploidentical HSCT using the Hopkins’ protocol [12] is very promising. This method has been further validated in a phase 2 trial [13]. In a multicentre trial (BMT CTN 1502), 31 patients received a haploidentical bone marrow transplant. Of these, 42% were reported as non-white. The 1-year overall survival was 81%. Acute GVHD grade II-IV was seen in 16% of subjects, and all cases were grade II. Chronic GVHD was seen in 26% of subjects at 1 year, and among these, subjects with at least 2 years of follow-up were off therapy. Target marrow cell dose for collection was 4 × 10^8^ nucleated marrow cells/kg of recipient ideal body weight, and a minimum of 2.5 × 10^8^ nucleated marrow cells/kg was recommended for a successful outcome and engraftment [13]. If the marrow dose is less than this minimum target, there is a very high risk of primary or secondary graft failure. In our cohort, patient #1 received less than the recommended dose, and the two others received more than 2.5 × 10^8^ nucleated marrow cells/kg. All patients were engrafted. 

Collecting an adequate cell dose by bone marrow harvesting can be especially challenging if there is a weight discrepancy between the donor and recipient. Another challenge arises if there is a major ABO blood group incompatibility between the donor and recipient. Such cases require red cell depletion of the bone marrow product, leading to a loss of stem cells. Moreover, if the final collection is below the target, another collection is not possible.

In these situations, the alternative is to use PBSC, as the cell dose collected is not dependent on donor weight, and ABO blood group compatibility is not an issue. The main adverse effect is a higher risk of chronic GVHD. As a general rule, bone marrow is the preferred graft source for hematopoietic stem cell transplantation in SAA. With the use of PBSC, there is a higher risk of chronic GVHD [19]. Nevertheless, in special situations, PBSC may be the preferred option, especially if collecting adequate bone marrow cells is likely to be unsuccessful.

At our centre, we occasionally prefer to use PBSC instead of BM when the collection of adequate cells by BM harvest is likely to be inadequate or when there is an urgency for neutrophil recovery to overcome a serious infection. At times, the donor is only willing to be subjected to PBSC collection. This approach worked well in patient #2 in our study, who received PBSCs and had no GVHD. In our earlier experience of using transplants in severe aplastic anemia, we used PBSCs as a graft source in 20.7% cases. The use of PBSCs was not a significant risk factor for overall survival or GVHD [20]. In an EBMT study on haploidentical transplants for aplastic anemia, there was no adverse effect of using PBSCs compared to BM as the graft source [11].

Haplo-identical transplants are complex and may have their own unique complications of low immune reconstitution and viral infections. One major concern is the very high risk of graft rejection in those who have a donor-specific antibody (DSA). It is important to test all patients for DSAs. If a DSA is present at a high level (general consensus at mean fluorescence intensity >1000 by solid phase immunoassay), the potential haploidentical donor should not be selected for transplant [13].

Our patients were all young, and some had infections prior to their transplant. They all had a rapid recovery of their neutrophils and platelets after the transplant procedure. Moreover, two patients became pregnant, and one patient’s menstrual cycle recovered. Hence, fertility may be preserved in some patients. This is a pilot study and should be considered as such, but based on our experience and the emerging literature, we feel a haplo-identical transplant can be offered to select young patients with SAA who have a low chance of response to conventional immunosuppressive therapy. Canadian centres should collaborate to share their experience of haploidentical transplants to improve the outcomes of patients with severe aplastic anemia.

## Figures and Tables

**Table 1:** Transplant Characteristics.

Patient Characteristics	#1	#2	#3	#4
Sex	F	F	F	F
Age in years at transplant	23	20	25	29
Ethnicity	South Asian	East Asian	Black	East Asian
Absolute Neutrophil count pre-transplant	0.0	5.0	0.3	0.3
Platelets count pre-transplant	7 × 10^9^/L	5 × 10^9^/L	3 × 10^9^/L	10 × 10^9^/L
Donor age/relationship	52/M, father	37/F/mother	59/mother	18/brother
Recipient/Donor Blood Group	O neg/A pos	AB pos/A pos	O pos/O pos	B pos/B pos
Recipient/Donor CMV	pos/pos	Pos/pos	Pos/pos	Pos/neg
Graft source	BM	PB	BM	BM
Cell dose	TNC 1.27 × 10^8^/kg (CD34 2.29 × 10^6^/kg)	CD34 8.4 × 10^6^/kg	TNC 2.7 × 10^8^/kg	TNC 4.27 × 10^8^/kg
Time from diagnosis to transplant	2 months	124 months	6 months	15 months
KPS	70	100	90	90
HCT-CI	3	0	0	1
Conditioning TBI dose	400cGy	200CGy	400cGy	400cGy
Day of neutrophil engraftment	22	17	20	22
Day of platelet engraftment	118	17	30	20
Discharge on day post-transplant	41	22	23	31
Day 30 chimerism	Complete	T cells 64%, myeloid 96%	Complete	T cells inconclusive, myeloid complete
Day 60 chimerism	Complete	Complete	Complete	Complete
Acute GVHD	Nil	Nil	Grade 2, skin	Nil
Chronic GVHD	Nil	Nil	Nil	Nil
Complications				
Bacteremia during transplant	Yes	No	Yes	Yes
CMV reactivation	Y	N	N	Y
EBV reactivation	Y	N	N	Y
BK virus hemorrhagic cystitis	Yes, Grade 1	N	Yes, Grade 4	N
Other complications	VOD. Poor graft function, treated with eltrombopag	HSV1	Hemorrhagic cystitis required hyperbaric Oxygen	Shingles one year post-transplant
Last follow-up	3 y and 9 m	3 y and 8 m	3 y and 1 m	2 y and 1 m
Chimerism (not repeated if blood counts are stable)	CD3 and CD 33: 100% at 9 m	CD3 96.8% and CD33 98.7% at 4 m	CD3 98.9% and CD33 97.7% at 7 m	CD3 95.1% and CD33 97.3% at 2 m
Ovarian function	No recovery	Normal menses	Pregnant and delivered a healthy baby (approx. 3 y after transplant)	Pregnant
Social	Continuing studies	Studying	Studying	Working

BM—bone marrow, PB—peripheral blood, TNC—total nucleated cells, KPS—Karnofsky performance score, HCT-CI—hematopoietic cell transplantation comorbidity index, TBI—total body irradiation, GVHD—graft versus host disease, VOD—veno-oclusive disease.

## Data Availability

Not available.

## References

[1] Killick S.B., Bown N., Cavenagh J., Dokal I., Foukaneli T., Hill A., Hillmen P., Ireland R., Kulasekararaj A., Mufti G. (2016). Guidelines for the diagnosis and management of adult aplastic anaemia. Br. J. Haematol..

[2] Young N.S. (2018). Aplastic Anemia. N. Engl. J. Med..

[3] McCahon E., Tang K., Rogers P.C., McBride M.L., Schultz K.R. (2003). The impact of Asian descent on the incidence of acquired severe aplastic anaemia in children. Br. J. Haematol..

[4] Kumar R., Kimura F., Ahn K.W., Hu Z.H., Kuwatsuka Y., Klein J.P., Pasquini M., Miyamura K., Kato K., Yoshimi A. (2016). Comparing Outcomes with Bone Marrow or Peripheral Blood Stem Cells as Graft Source for Matched Sibling Transplants in Severe Aplastic Anemia across Different Economic Regions. Biol. Blood Marrow Transplant..

[5] Frickhofen N., Heimpel H., Kaltwasser J.P., Schrezenmeier H., German Aplastic Anemia Study Group (2003). Antithymocyte globulin with or without cyclosporin A: 11-year follow-up of a randomized trial comparing treatments of aplastic anemia. Blood.

[6] Georges G.E., Doney K., Storb R. (2018). Severe aplastic anemia: Allogeneic bone marrow transplantation as first-line treatment. Blood Adv..

[7] Marsh J.C.W., Risitano A.M., Mufti G.J. (2019). The Case for Upfront HLA-Matched Unrelated Donor Hematopoietic Stem Cell Transplantation as a Curative Option for Adult Acquired Severe Aplastic Anemia. Biol. Blood Marrow Transplant..

[8] Dehn J., Buck K., Maiers M., Confer D., Hartzman R., Kollman C., Schmidt A.H., Yang S.Y., Setterholm M. (2015). 8/8 and 10/10 high-resolution match rate for the be the match unrelated donor registry. Biol. Blood Marrow Transplant..

[9] Xu L.P., Wang S.Q., Wu D.P., Wang J.M., Gao S.J., Jiang M., Wang C., Zhang X., Liu Q., Xia L. (2016). Haplo-identical transplantation for acquired severe aplastic anaemia in a multicentre prospective study. Br. J Haematol..

[10] Lu Y., Sun R.J., Zhao Y.L., Xiong M., Cao X.Y., Zhang J.P., Wei Z.-J., Zhou J.-R., Liu D.Y., Lu D.-P. (2018). Unmanipulated Haploidentical Hematopoietic Stem Cell Transplantation Achieved Outcomes Comparable with Matched Unrelated Donor Transplantation in Young Acquired Severe Aplastic Anemia. Biol. Blood Marrow Transplant..

[11] Prata P.H., Eikema D.J., Afansyev B., Bosman P., Smiers F., Diez-Martin J.L., Arrais-Rodrigues C., Koc Y., Poiré X., Sirvent A. (2020). Haploidentical transplantation and posttransplant cyclophosphamide for treating aplastic anemia patients: A report from the EBMT Severe Aplastic Anemia Working Party. Bone Marrow Transplant..

[12] DeZern A.E., Zahurak M.L., Symons H.J., Cooke K.R., Rosner G.L., Gladstone D.E., Huff C.A., Swinnen L.J., Imus P., Borrello I. (2020). Haploidentical BMT for severe aplastic anemia with intensive GVHD prophylaxis including posttransplant cyclophosphamide. Blood Adv..

[13] DeZern A.E., Eapen M., Wu J., Talano J.A., Solh M., Dávila Saldaña B.J., Karanes C., Horwitz M.E., Mallhi K., Arai S. (2022). Haploidentical bone marrow transplantation in patients with relapsed or refractory severe aplastic anaemia in the USA (BMT CTN 1502): A multicentre, single-arm, phase 2 trial. Lancet Haematol..

[14] de Latour R.P., Kulasekararaj A., Iacobelli S., Terwel S.R., Cook R., Griffin M., Halkes C.J.M., Recher C., Barraco F., Forcade E. (2022). Eltrombopag Added to Immunosuppression in Severe Aplastic Anemia. N. Engl. J. Med..

[15] Tiercy J.M. (2016). How to select the best available related or unrelated donor of hematopoietic stem cells?. Haematologica.

[16] Patel B.A., Groarke E.M., Lotter J., Shalhoub R., Gutierrez-Rodrigues F., Rios O., Raffo D.Q., Wu C.O., Young N.S. (2022). Long-term outcomes in patients with severe aplastic anemia treated with immunosuppression and eltrombopag: A phase 2 study. Blood.

[17] de Latour R.P., Brunstein C.G., Porcher R., Chevallier P., Robin M., Warlick E., Xhaard A., Ustun C., Larghero J., Dhedin N. (2013). Similar Overall Survival Using Sibling, Unrelated Donor, and Cord Blood Grafts after Reduced-Intensity Conditioning for Older Patients with Acute Myelogenous Leukemia. Biol. Blood Marrow Transplant..

[18] de Latour R.P., Chevret S., Jubert C., Sirvent A., Galambrun C., Ruggeri A., Gandemer V., Cornillon J., Rialland F., Dalle J.-H. (2018). Unrelated cord blood transplantation in patients with idiopathic refractory severe aplastic anemia: A nationwide phase 2 study. Blood.

[19] Bacigalupo A., Socie G., Schrezenmeier H., Tichelli A., Locasciulli A., Fuehrer M., Risitano A.M., Dufour C., Passweg J.R., Oneto R. (2012). Bone marrow versus peripheral blood as the stem cell source for sibling transplants in acquired aplastic anemia: Survival advantage for bone marrow in all age groups. Haematologica.

[20] Salas M.Q., Atenafu E.G., Lam W., Law A.D., Kim D.D.H., Michelis F.V., Al-Shaibani Z., Gerbitz A., Lipton J.H., Viswabandya A. (2020). High Overall and GVHD-Free Survival in Patients with Aplastic Anemia Receiving in vivo T-cell Depletion Transplants and Long-Term Complications. Blood Cell Ther..

